# Childhood maltreatment and leukocyte telomere length in men and women with chronic illness: an evaluation of moderating and mediating influences

**DOI:** 10.1017/S0033291722003543

**Published:** 2023-10

**Authors:** Alexandra Connor, Louisia Starnino, Lambert Busque, Jean-Claude Tardif, Vincent Bourgoin, Marie-Pierre Dubé, David Busseuil, Bianca D'Antono

**Affiliations:** 1 Research Centre, Montreal Heart Institute, Montreal, Canada; 2 Psychology Department, Université de Montréal, Montreal, Canada; 3 Psychology Department, Université du Québec à Montréal, Montreal, Canada; 4 Hematology Division, Research Center, Hôpital Maisonneuve-Rosemont; Université de Montréal, Montreal, Canada; 5 Department of Medicine, Université de Montréal, Montreal, Canada

**Keywords:** Anxiety, childhood maltreatment, chronic illness, coronary artery disease, depression, emotional dysregulation, leukocyte telomere length, perceived stress

## Abstract

**Background:**

Childhood maltreatment can result in lifelong psychological and physical sequelae, including coronary artery disease (CAD). Mechanisms leading to increased risk of illness may involve emotional dysregulation and shortened leukocyte telomere length (LTL).

**Methods:**

To evaluate whether (1) childhood maltreatment is associated with shorter LTL among older adults with CAD or other chronic illnesses; (2) sex and/or CAD status influence these results; and (3) symptoms of anxiety, depression, and stress moderate or mediate the association between childhood maltreatment and LTL, men and women (*N* = 1247; aged 65 ± 7.2 years) with and without CAD completed validated questionnaires on childhood maltreatment, symptoms of depression, anxiety, and perceived stress. LTL was measured using quantitative polymerase chain reaction. Analyses included bivariate correlations, hierarchical regressions, and moderation/mediation analyses, controlling for sociodemographic and lifestyle variables.

**Results:**

Childhood maltreatment was associated with significantly shorter LTL (*r* = −0.059, *p* = 0.038, *b* = −0.016, *p* = 0.005). This relation was not moderated by depression, anxiety, nor perceived stress, though there was mitigated evidence for absence of a maltreatment-LTL relation in men with CAD. Stress perception (but not anxiety or depression) partially mediated the relation between childhood maltreatment and LTL [Indirect effect, *b* = −0.0041, s.e. = 0.002, 95% CI (−0.0085 to −0.0002)].

**Conclusions:**

Childhood maltreatment was associated with accelerated biological aging independently of patient characteristics. Emotional dysregulation resulting in chronic stress may contribute to this process. Whether stress management or other interventions may help prevent or slow premature aging in those who have suffered maltreatment requires study.

The aftermath of childhood maltreatment, involving sexual, physical, emotional abuse, or neglect, can be devastating and result in serious lifelong psychological, interpersonal, and physical consequences (Burczycka & Conroy, 2017; Gilbert et al., 2009). For example, survivors of childhood maltreatment are at a higher risk of developing anxiety and mood disorders or symptoms (Li, D'Arcy, & Meng, 2016), report more stress (Bossé, Stalder, & D'Antono, 2018; Garami et al., 2019; LoPilato et al., 2020), and are at an increased risk for developing chronic physical ailments such as coronary artery disease (CAD) (Jakubowski, Cundiff, & Matthews, 2018). Mechanisms leading to increased risk of CAD or other chronic physical conditions are likely diverse and may involve emotional dysregulation and biological changes at the cellular level, such as in leukocyte telomere length (LTL).

Telomeres are non-coding DNA sequences on the ends of chromosomes that are meant to protect the coding sequences of DNA from potential damage that may occur during the cell division process (Blackburn, 2001; Blasco, 2005). Because of their incomplete replication and progressive shortening over time (Blackburn, 2000), telomere length (TL) has been suggested as a marker of biological aging (Terry, Nolan, Andersen, Perls, & Cawthon, 2008; Zglinicki & Martin-Ruiz, 2005). Once telomeres reach a critical length, affected cells may fail to operate normally or die, contributing to disease development (Brouilette et al., 2007; Nilsson, Dahlman, Roos, Nordfjäll, & Melander, 2011; Samani & van der Harst, 2008). Importantly, a growing body of research suggests that exposure to childhood maltreatment is associated with shorter TL.

Three meta-analyses reported small but significant relations between childhood trauma or adversity and TL in adulthood (Bürgin et al., 2019; Li, He, Wang, Tang, & Chen, 2017; Ridout et al., 2018). Considerable heterogeneity was observed, which the authors attributed to potential individual differences, including sex, health status, and characteristics of the studies, such as sample size and included covariates. For example, Bürgin et al. (2019) observed that results from larger studies were more heterogenous, which they hypothesized was due to a greater statistical control of variables that may actually moderate or mediate the relationship. Controlling for them may thus conceal associations of childhood maltreatment with TL.

There is ample reason to believe that individual differences may exist. Men and women, for example, differ in the types of traumas they may have been exposed to (Burczycka & Conroy, 2017; Gilbert et al., 2009) and data suggests that men generally have shorter TL, and faster attrition rates compared to women (for a review see Barrett and Richardson, 2011). Although Bürgin et al. (2019) did not find sex differences in the relation between childhood maltreatment and TL, few studies included in their meta-analysis actually examined sex differences directly.

On the other hand, studies that included individuals with medical illnesses showed smaller effect sizes than those with healthy individuals, suggestive of a potential moderating effect of physical health status (Ridout et al., 2018). Given the poorer prognosis related to shorter LTL in individuals with chronic illnesses (Farzaneh-Far et al., 2008; Opstad, Kalstad, Pettersen, Arnesen, & Seljeflot, 2019; Tian et al., 2017; Willeit et al., 2010), and the association between child maltreatment and the development of chronic illnesses (Hughes et al., 2017; Maschi, Baer, Morrissey, & Moreno, 2013), examination of the impact of health status, especially CAD, on the relation between childhood maltreatment and LTL appears warranted. Indeed, there are high prevalence, morbidity, and mortality rates of CAD compared to most other chronic illnesses (Statistics Canada, 2020), as well as differences in psychological distress in CAD compared to non-CAD individuals (e.g. Vaillancourt, Busseuil, and D'Antono, 2021). Thus, the relation between childhood maltreatment and LTL may very well differ as a function of presence of CAD *v.* other, less fatal chronic illnesses.

The relation between childhood maltreatment and LTL was also weaker in studies whose samples were composed of individuals suffering from psychiatric conditions compared to studies whose samples were non-clinical (Bürgin et al., 2019; Ridout et al., 2018). To our knowledge, the only two studies that investigated whether depression influenced the relation between childhood maltreatment and LTL found no evidence of moderation in 92 young Colombians (Jiménez, Pereira-Morales, Adan, & Forero, 2019) nor in 180 middle-aged Ukrainians (Vincent et al., 2017). Data concerning moderation by other markers of psychological distress, such as anxiety or stress is lacking.

While psychological distress may relate to the individuals' recent life situations, it may also reflect emotional dysregulation resulting from childhood maltreatment experiences (Bowlby, 1979; Neacsiu, Bohus, & Linehan, 2014; Porges, 2009; Thayer & Lane, 2000), and associated symptoms of complex post-traumatic stress disorder (Herman, 1992). These children may develop emotional reactivity and difficulties implementing strategies to regulate it (i.e. emotional dysregulation; Gross and Jazaieri, 2014), including heightened fight-flight or freeze responses and behaviors (e.g. substance use, overeating, social withdrawal) that may persist over a lifetime, and increase their risk for premature aging and illness (Ellis, Bianchi, Griskevicius, & Frankenhuis, 2017; McEwen & Stellar, 1993; Porges, 2009; Thayer & Lane, 2000). While shorter TL have been observed in individuals with symptoms or disorders of depression (Ridout, Ridout, Price, Sen, & Tyrka, 2016), anxiety (Malouff & Schutte, 2017), and stress (Mathur et al., 2016), research on whether psychological distress mediates the childhood maltreatment-LTL relation is extremely limited. One study found that the self-control aspect of self-regulation (the ability to manage emotions in order to conduct behavior) partially mediated the relationship between adverse childhood adversity and TL in a sample of 2527 children with unwed parents, though the effect was very small (*b* = −0.002) (Sosnowski et al., 2020). In another large investigation in middle-aged men, 16% of the effect of stressful childhood events on LTL was mediated by depressive mood (Osler, Bendix, Rask, & Rod, 2016).

Thus, evidence suggests that childhood maltreatment is associated with shorter LTL, and preliminary data suggests that characteristics of individuals may moderate or mediate this relation. The objectives of the study are therefore to replicate and extend findings regarding childhood maltreatment and LTL in older men and women with CAD or other chronic non-cardiovascular disease (CVD) and determine whether sex and CAD status moderate the observed relations. Finally, we seek to determine whether correlates of emotional dysregulation (anxiety, depression, stress) moderate or cross-sectionally mediate this relationship. It is hypothesized that self-reported childhood maltreatment will be associated with shorter LTL. Given the paucity of information on potential moderation or mediation of this relation, no directional hypotheses are presented at this time.

## Methods

This study is part of an ongoing prospective research project (BEL-AGE) examining the role of psychological burden in pathological aging. This study was approved by the Research Ethics and New Technology Development Committee of the Montreal Heart Institute (#11-1313).

## Participants

A total of 1325 (40% women) participants with CAD or other non-CVD illnesses (mean age = 65.4 ± 7.2 years) were recruited from the André and France Desmarais Hospital Cohort of the Montreal Heart Institute (the MHI cohort). Participants were eligible for the BEL-AGE study if they met the following criteria when they first enrolled into the MHI cohort: they were (a) aged between 30 and 70 years old, (b) living in the greater Montreal area, (c) spoke English or French, (d) had no previous or current diagnosis of serious psychological disorders (e.g. bipolar disorder, schizophrenia, dementia) or major cognitive impairment that could hinder the participants understanding of any aspect of the study, (e) had no previous or current diagnosis of major life-threatening diseases other than CAD (e.g. AIDS, cancer, amyotrophic lateral sclerosis, Creutzfelt–Jakob disease), (f) not pregnant or breastfeeding (g) or if none of their family members or spouse had previously participated in BEL-AGE, or were scheduled to participate. Depression, anxiety, and endocrine disorders were not excluded. Skin cancer was not excluded because of its high prevalence and benign course with an early diagnosis. CAD was defined as having previously suffered at least one myocardial infarction, coronary artery bypass, coronary angioplasty, or stenosis of more than 50% on an angiography. An absence of CAD, angina, arrhythmia, congenital heart disease, heart failure, cardiomyopathy, and stroke was required for non-CVD status. Those who were considered healthy had no notable or chronic health issue. Medical history was self-reported and verified with the participants' medical file in the case of CAD. A total of seventy-eight participants were excluded from the current study analyses either because they did not meet the inclusion criteria (*n* = 22), had missing LTL or psychological data (*n* = 26), or were suffering from no health conditions (*n* = 30), resulting in a final sample of 1247 individuals.

## Procedure

Potential participants were contacted by telephone and screened for eligibility. Appointments were scheduled on weekdays between 8:00 and 10:00 a.m. in order to control for circadian rhythms. Except for water and prescribed medication, participants were asked to refrain from eating and drinking 12 h before their appointment and using illicit substances or alcohol 24 h before their appointment. Participants who did not adhere to these requirements or presented with colds or other acute symptoms were rescheduled. After written consent was provided, anthropomorphic data (weight, height, and waist circumference) were measured, and a 35 mL sample of blood was obtained. Participants were questioned regarding their demographic and medical information, underwent a cognitive screen for other purposes, and completed several questionnaires. Only their travel/parking costs were reimbursed.

## Measures

### Sociodemographic and health variables

Data on sex, age, ethnicity, years of schooling, civil status, personal and family income, personal and family medical history were obtained, as was information on the participant's health behaviors (tobacco use, alcohol, caffeine consumption, diet, and physical activity). The participants provided a current list of medications.

*The Childhood Trauma Questionnaire* (CTQ; Bernstein et al., 1994; Paquette, Laporte, Bigras, and Zoccolillo, 2004) was used to assess childhood and adolescent maltreatment. This questionnaire is comprised of 25 items with 5 subscales including: physical, sexual, and emotional abuse, as well as physical and emotional neglect. Each item uses a five-point scale ranging from ‘never true’ to ‘very often true’. The sum of scores is calculated and participants are also categorized as having experienced childhood maltreatment when they have a score within the moderate-to-severe range on at least one subscale: emotional abuse (≥13), physical abuse (≥10), sexual abuse (≥8), emotional neglect (≥15), and physical neglect (≥10). This questionnaire has high internal consistency (*α* = 0.89) and good test-retest reliability over a 4-month period in both English and French versions (Bernstein & Fink, 1998; Paquette et al., 2004). In the current sample, overall internal consistency was excellent (*α* = 0.91) and ranged from 0.82 (emotional abuse, physical abuse, sexual abuse, and emotional neglect) to 0.58 for the physical neglect subscale, consistent with previous studies (Dekker et al., 2005; Ekelund et al., 2006).

*The Center of Epidemiological Studies Depression Scale Revised* (CES-D-R; Van Dam and Earleywine, 2011), measures the presence of depressive symptoms over the past 2 weeks on a likert scale from ‘not at all or less than one day’ to ‘nearly every day for 2 weeks’. Consisting of 20 items, total scores range from 0 to 60, with scores higher than or equal to 16 indicating greater risk of clinical depression. This questionnaire has excellent internal consistency (*α* > 0.85) for both English (Van Dam & Earleywine, 2011) and French versions (Caron, 2001). Test-retest reliability over 3–6 months is 0.54–0.59 for the English (Radloff, 1977; Roberts, 1980) and French (Caron, 2001) versions, respectively.

*State-Trait Anxiety Inventory-state version*. (STAI; Spielberger, Gorsuch, Lushene, Vagg, and Jacobs, 1983) is a 20-item questionnaire assessing participant's current state of anxiety. Participants responded to each item with a 4-point Likert scale ranging from ‘not at all’ to ‘very much’. Total scores range from 20–80, while a score of 40 or greater is considered clinically elevated. This questionnaire has excellent internal consistency (average *α* = 0.89) and a test-retest reliability over a period of 3.5 months of 0.75 (Spielberger, Gonzalez-Reigosa, Martinez-Urrutia, Natalicio, & Natalicio, 1971; Spielberger et al., 1983). Similar values were found for the French-Canadian version (Gauthier & Bouchard, 1993).

*The Perceived Stress Questionnaire* (PSQ; Levenstein et al., 1993). Consisting of 30 items, this questionnaire, specifically designed for psychosomatic research, covers seven dimensions including: harassment, overload, irritability, lack of joy, fatigue, worries, and tension. It is used to measure perceived stressful life events, circumstances, and reactions to stress. Participants rate how frequently they experienced each of the stress-related statements over the past 2 years on a four-point scale ranging from ‘almost never’ to ‘usually’. It is scored using the sum of the individual items, as well as the PSQ index (raw score-30)/90), which represents the severity of stress of each participant, varying from 0 (lowest level of stress) to 1 (highest level of stress). A score of 0.45–0.60 on the PSQ index indicates a moderate stress level, while scores >0.60 reflect high stress. This questionnaire has excellent internal consistency for the English version (*α* = 0.90), and a test-retest reliability over a period of 6 months of 0.82 (Lehman, Burns, Gagen, & Mohr, 2012; Levenstein et al., 1993; Montero-Marin, Piva Demarzo, Pereira, Olea, & García-Campayo, 2014), with similar values for the French version (Consoli, Taine, Szabason, Lacour, & Metra, 1997).

We have shown in previous research that the psychometric properties for each of these psychological variables within our sample are consistent with the existing literature (Vaillancourt et al., 2021).

### Leukocyte telomere length

Quantitative polymerase chain reaction (qPCR) was used to measure TL, using the modified method (Epel et al., 2004) of the protocol described by Cawthon (2002) as previously reported in Starnino et al. (2021). This method is based on determining the number of telomere DNA sequences (*T*) on the number of copies of a control gene (RPLP0, 60S acidic ribosomal protein P0) (*S*). Briefly, DNA was extracted from blood leukocytes using BioRobot M48 system (Qiagen, Germany). Serial dilutions of a reference DNA from a single normal individual were made to establish a standard curve (mean efficiency for *T* = 120% and *S* = 102%). The *T*/*S* was normalized to 1 and used to calculate the *T*/*S* ratio for each sample of DNA tested. The relative ratio of a sample thus represents the number of copies of telomeres relative to the reference sample. All qPCR was performed on the ViiA™7 real-time PCR system (Applied Biosystems, Massachusetts, USA) and with the QuantStudio analysis software (Applied Biosystems, Massachusetts, USA). All samples were measured in triplicate, and their mean was used for analyses. If the standard deviation of cycle threshold (Ct s.d.) was over 0.3, the outlier was omitted, and duplicate was considered. Otherwise, the sample was repeated.

## Statistical analyses

### Preliminary analyses

Data analysis was performed using IBM SPSS, Version 26. Moderation and mediation analyses were performed using the computational tool PROCESS version 3.5 (Hayes, 2020). Log-transformations were applied to depressive symptoms and anxiety data, while square root transformations were applied to childhood maltreatment data to normalize their distributions.

Covariates, including age, hours of exercise per week, civil status, years of education, smoker status, metabolic triad (diagnosis of hypercholesterolemia, hypertriglyceridemia, and /or essential hypertension), body mass index (BMI), and family income, were chosen based on the existing literature on childhood maltreatment and LTL (Bürgin et al., 2019; Ridout et al., 2018).

### Main analyses

Bivariate correlations were conducted to examine the relationship between childhood maltreatment and LTL. To examine the association between childhood maltreatment and LTL controlling for covariates, a hierarchical linear regression was conducted with the covariates as well as sex and CAD-status variables in block 1, and childhood maltreatment in block 2.

Moderation analyses were performed to verify whether sex and/or CAD-status moderated the association between childhood maltreatment and LTL, controlling for other covariates. Similar analyses were conducted to verify whether depression, anxiety, and stress independently moderated the relation between childhood maltreatment and LTL. The 2- and 3-way interactions of childhood maltreatment with sex, CAD status, and/or psychological variables in moderation analyses were constructed from centered variables. If the interactions terms were not significant, they were removed from the equation and the more parsimonious model(s) were retained. Significant interactions were explored via simple slope analyses.

A parallel mediation analysis was conducted to investigate whether depression, anxiety, and stress (entered simultaneously) mediated the relationship between childhood maltreatment and LTL, controlling for covariates, including sex and CAD status. The indirect effects were tested using non-parametric bootstrapping, with effects considered statistically significant when 0 falls outside the 95% confidence intervals. Bootstrapping was performed using 5000 samples.

A two-sided *p* value < 0.05 was considered statistically significant for main effects, and interactions meeting a *p* value < 0.10 were examined to avoid type II errors (Winer, 1962). Main analyses were conducted using continuous measures of CTQ, depression, anxiety, and stress.

## Results

Demographic characteristics of men and women, and individuals with and without CAD are presented in Table 1. While childhood maltreatment did not differ as a function of sex nor CAD status, women had significantly longer LTL compared to men, and non-CVD individuals had significantly longer LTL than those with CAD. Furthermore, women reported significantly more depression, anxiety or stress compared to men.

**Table 1.** Participant characteristics (Mean ± s.d.)

	CAD (*n* = 668)	Non CVD (*n* = 579)	Women (*n* = 491)	Men (*n* = 756)	All participants (*N* = 1247)
Demographic variables					
Age, years	66.28 (6.53)	64.70 (7.38)	65.66 (7.14)	65.47 (6.88)	65.55 (6.99)
Race *N* (%)					
*Caucasian*	661 (99.0%)	567 (98.1%)	485 (99.0%)	743 (98.3%)	1228 (98.6%)
First spoken language *N* (%)					
*French*	626 (93.7%)	522 (90.2%)	455 (92.7%)	693 (91.7%)	1148 (92.1%)
*English*	13 (1.9%)	15 (2.6%)	9 (1.8%)	19 (2.5%)	28 (2.2%)
Civil status *N* (%)					
*Single*	55 (8.2%)	59 (10.2%)	54 (11.0%)	60 (7.9%)	114 (9.1%)
*Civil union/married*	483 (72.3%)	429 (74.1%)	325 (66.2%)	587 (77.6%)	912 (73.1%)
*Separated/divorced/widowed*	130 (19.5%)	91 (15.7%)	112 (22.8%)	109 (14.4%)	221 (17.7%)
Years of education	13.83 (3.67)	14.82 (3.47)	13.93 (3.53)	14.51 (3.65)	14.29 (3.62)
Employment status *N* (%)					
*Full-time*	107 (16.0%)	140 (24.2%)	80 (16.3%)	167 (22.1%)	274 (19.8%)
*Part-time*	63 (9.4%)	62 (10.7%)	55 (11.2%)	70 (9.3%)	125 (10.0%)
*Retired*	488 (73.1%)	373 (64.4%)	346 (70.5%)	515 (68.1%)	861 (69.0%)
Annual family income *N* (%)					
≤ *$29 999*	95 (14.3%)	46 (8.0%)	73 (15.1%)	68 (9.0%)	141 (11.4%)
*$30 000–$59 999*	230 (34.6%)	194 (33.8%)	196 (40.4%)	228 (30.3%)	424 (34.0%)
*$60 000–$99 999*	168 (25.3%)	144 (25.1%)	119 (24.5%)	193 (25.6%)	312 (25.2%)
≥ *$100 000*	171 (25.8%)	190 (33.1%)	97 (20.0%)	264 (35.1%)	361 (29.2%)
Health behaviors					
Exercice, hours/week	2.82 (3.30)	3.31 (3.34)	2.96 (3.06)	3.10 (3.49)	3.04 (5.32)
Body mass index, kg/m^2^	29.62 (4.97)	28.78 (5.31)	28.64 (5.83)	29.61 (4.62)	29.23 (5.15)
Current smoker *N* (%)	94 (14.1%)	33 (5.7%)	40 (8.1%)	87 (11.5%)	127 (10.2%)
Cigarettes/day	14.74 (10.36)	12.12 (8.77)	12.08 (7.19)	14.97 (10.98)	14.06 (10.00)
Psychotropic medication *N* (%)	159 (23.8%)	122 (21.1%)	144 (29.3%)	137 (18.1%)	281 (22.5%)
*Telomere length* (*T/S ratio*)	0.85 (0.18)	0.91 (0.20)	0.91 (0.20)	0.85 (0.18)	0.88 (0.19)
Psychological distress					
Depressive symptoms	7.05 (8.08)	6.35 (7.33)	7.25 (7.54)	6.38 (7.86)	6.72 (7.74)
Anxiety symptoms	29.17 (7.99)	29.05 (7.11)	29.96 (7.61)	28.56 (7.53)	29.11 (7.59)
Perceived stress	55.55 (13.77)	56.65 (14.60)	59.07 (14.50)	54.11 (13.61)	56.06 (14.17)
Childhood maltreatment					
Total score	36.76 (12.93)	35.80 (12.17)	37.22 (14.23)	35.73 (11.37)	36.32 (12.58)
Participants who experienced maltreatment (%)	227 (34.0%)	172 (29.7%)	166 (33.8%)	233 (30.8%)	399 (32.0%)
Number of maltreatment types experienced *N* (%)					
*No maltreatment*	441 (66.0%)	407 (70.3%)	325 (66.2%)	523 (69.2%)	848 (68.0%)
*One type*	120 (18.0%)	92 (15.9%)	84 (17.1%)	128 (16.9%)	212 (17.0%)
*Two types*	59 (8.8%)	34 (5.9%)	34 (6.9%)	59 (7.8%)	93 (7.5%)
*Three types*	18 (2.7%)	25 (4.3%)	23 (4.7%)	20 (2.6%)	43 (3.4%)
*Four types*	13 (1.9%)	11 (1.9%)	11 (2.2%)	13 (1.7%)	24 (1.9%)
*Five types*	17 (2.5%)	10 (1.7%)	14 (2.9%)	13 (1.7%)	27 (2.2%)

*Note*: CVD, cardiovascular disease.

### Childhood maltreatment and LTL are associated

Childhood maltreatment correlated with significantly shorter LTL (*r* = −0.059, *p* = 0.038). Table 2 presents the detailed results of the hierarchical linear regression controlling for covariates, in which the relationship between childhood maltreatment and LTL remained significant.

**Table 2.** Hierarchical regression analysis details for TL

	*b*	*t*	*p*	95% CI		Semipartial *r*
				LL	UL	
Block 1						
Age, years	−0.008	−9.74	<0.001	−0.009	−0.006	−0.261
Male *v.* female sex	−0.043	−3.6	<0.001	−0.066	−0.020	−0.098
CAD *v.* other chronic disease	−0.021	−1.78	0.075	0.002	−0.045	−0.048
Metabolic triad (yes *v.* no)	−0.017	−1.06	0.290	−0.050	0.015	−0.028
BMI, kg/m^2^	−0.002	−1.93	0.054	−0.004	<0.001	−0.052
Smoker (yes *v.* no)	−0.021	−1.17	0.244	−0.055	0.014	−0.031
Exercise, hours/week	0.002	1.33	0.184	−0.001	0.005	0.036
Civil status	−0.016	−2.27	0.024	−0.030	−0.002	−0.061
Years of education	0.002	1.24	0.214	−0.001	0.005	0.033
Family income	−0.010	−1.56	0.118	−0.021	0.002	−0.042
*F*(10, 1226) = 16.05 *p* < 0.001, *R*^2^ = 0.116, *R*^2^_adj_ = 0.109						
Block 2						
Childhood maltreatment	−0.016	−2.80	0.005	−0.027	−0.005	−0.075
*F*(1, 1225) = 15.39 *p* < 0.001, *R*^2^ = 0.121, *R*^2^_adj_ = 0.114, *R*^2^_add_ = 0.006						

*b*, estimated value of unstandardized regression coefficient; *t*, student's *t* distribution; LL, lower limit; UL, upper limit.

*Note*: Metabolic triad refers to the presence of hypercholesterolemia, hypertriglyceridemia, and/or essential hypertension.

### The relation between childhood maltreatment and LTL is not moderated by examined variables

The relation between childhood maltreatment and LTL was not moderated by sex (*p* = 0.896), CAD status (*p* = 0.971), nor by depression (*p* = 0.470), anxiety (*p* = 0.448), and stress (*p* = 0.673).

### Chronic stress mediates the relation between childhood maltreatment and LTL

**Fig. 1.**
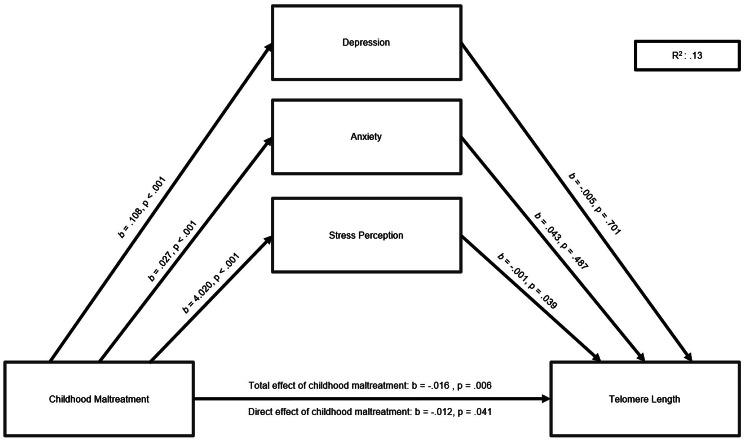
Mediation of the relation between childhood maltreatment and TL. A parallel mediation analysis revealed a significant indirect effect of childhood maltreatment on TL through stress perception [*b* = −0.0041, 95% CI (−0.0085 to −0.0002)].

Results of the parallel mediation analysis revealed that while childhood maltreatment predicted significantly higher levels of depression, anxiety, and stress (all *ps* < 0.001), only stress partially and significantly mediated the relationship between childhood maltreatment and LTL [Indirect effect – IE_PSQ_ = −0.0041, s.e. = 0.002, 95% CI (−0.0085 to −0.0002)], controlling for the specified covariates. The total (*b* = −0.016, *p* = 0.006) and direct (*b* = −0.012, *p* = 0.041) effects of childhood maltreatment on LTL were both negative and statistically significant. Results are depicted in Fig. 1.

### Post-hoc analyses

**Fig. 2.**
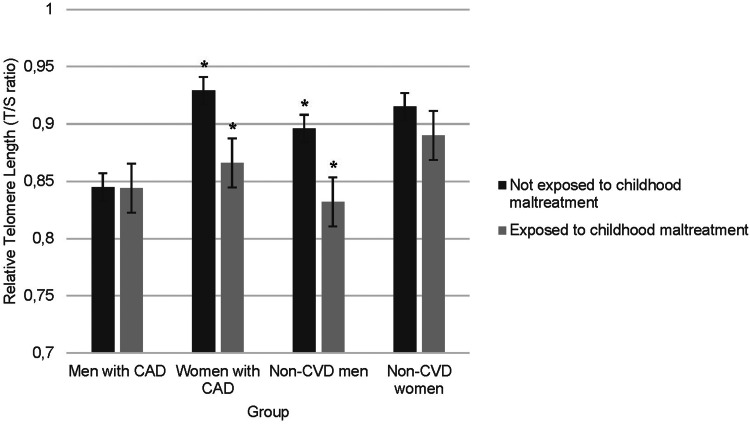
Group differences in relative telomere length as a function of sex, CAD status, and childhood maltreatment. Childhood maltreatment was associated with significantly shorter TL in women with CAD (*p* = 0.041) and non-CVD men (*p* = 0.016), and a trend towards the same in non-CVD women (*p* = 0.272). In men with CAD, there was no relation (*p* = 0.954). Significant differences between those exposed to childhood maltreatment and those not exposed are identified with asterisks within the figure.

A three-way (Sex by CAD-Status by Childhood Maltreatment) ANCOVA was conducted with LTL as dependent variable and childhood maltreatment as a dichotomous variable (based on CTQ cut-off) to examine whether comparing more severe to no/less severe maltreatment altered results. Significant main effects of childhood maltreatment [*F*(1,1221) = 10.10, *p* = 0.002] and sex [*F*(1,1221) = 13.09, *p* < 0.001], as well as a 3-way CAD-status by Sex by Childhood maltreatment interaction [*F*(1,1221) = 4.425, *p* = 0.036] were observed. More specifically, childhood maltreatment was associated with significantly shorter LTL in women with CAD (*p* = 0.041) and in non-CVD men (*p* = 0.016), with a trend towards the same in non-CVD women (*p* = 0.272). In men with CAD, there was no relation (*p* = 0.954) (see Fig. 2).

Bivariate correlations between LTL and the number of maltreatment subtypes (number of CTQ subscales for which each person surpassed the cut-off) were performed to examine whether exposure to more types of childhood maltreatment was associated with shorter LTL. Number of maltreatment subtypes was associated with significantly shorter LTL (*r* = −0.059, *p* = 0.038).

## Discussion

The current project evaluated the association between childhood maltreatment and LTL among older men and women with CAD or other non-CVD illness, and whether individual characteristics (sex, CAD status, and correlates of emotional dysregulation) of participants moderated and/or mediated this relation. Findings confirmed that greater maltreatment in childhood was associated with significantly shorter LTL. None of the individual characteristics examined moderated this relation when childhood maltreatment was examined as a continuous variable. However, when childhood maltreatment was dichotomized to capture more severe maltreatment, men with CAD did not show the expected association. Perceived chronic stress partially and significantly mediated the relation between maltreatment experiences and later LTL. Findings were independent of sociodemographic, medical, and lifestyle variables.

That individuals with childhood maltreatment had shorter LTLs in the current study is consistent with the results of previous meta-analyses (Bürgin et al., 2019; Li et al., 2017; Ridout et al., 2018). In contrast to Li et al. (2017), however, our finding remained significant after adjusting for age (and other covariates with the potential to impact on LTL). While robust, the effect accounted for only 0.6% of the variance in LTL. This may seem small, but the association was actually larger than the other variables included, with the exception of age and sex. Furthermore, childhood maltreatment was associated with up to 5 years greater biological age compared to those who were not exposed to childhood maltreatment (see online Supplementary Table S1 for the calculation of biological years difference from *T*/*S* ratio). Nonetheless, overall, only 12.6% of variance in LTL was explained by the model, indicating need for continued research.

The few studies that examined sex differences in the relation between childhood maltreatment and TL reported similar effects of maltreatment in men and women (Bürgin et al., 2019). This was consistent with our own results using a continuous measure of childhood maltreatment. Thus, while boys and girls differ in the types of maltreatment they are more likely to be exposed to (Burczycka & Conroy, 2017), it may be the experience of any severe maltreatment that contributes to premature aging, though exposure to a greater number of maltreatment types may be more damageable as suggested by this and other research (Mayer et al., 2019; Ridout et al., 2015; Surtees et al., 2012; Theall, Brett, Shirtcliff, Dunn, & Drury, 2013).

According to one meta-analysis (Bürgin et al., 2019), childhood trauma was more weakly associated with TL in investigations that included individuals suffering from medical conditions suggesting that health status may moderate the relation. To our knowledge, only one prior study specifically examined this issue (Malan-Müller et al., 2013). Although they found that individuals with HIV had significantly shorter LTL than those without, HIV status did not moderate the association between maltreatment and LTL. This is consistent with our own results concerning CAD status. It is noteworthy that the post hoc analyses using a categorical approach to measure childhood maltreatment did find health status to influence the association as a function of participants' sex. Indeed, it was particularly among the women with CAD and men (and to a lesser degree, women) with non-cardiovascular chronic disease that childhood maltreatment was found to be associated with the greatest telomere attrition. Why men with CAD did not show the same pattern is unclear. One possible explanation may relate to survivor bias. Results from a meta-analysis suggest that psychological distress (that is greater in survivors of childhood maltreatment) may be associated with a slightly but significantly poorer prognosis in men with CAD as compared to women (Smaardijk, Maas, Lodder, Kop, & Mommersteeg, 2020). If this is the case, it may be that those men with CAD who were able to participate in the study were more resilient for some reason to the effects of adversity, in contrast to others who may have already passed away or been too ill to participate, in effect concealing the true impact of maltreatment among these men. In addition, shorter telomeres have been observed in individuals with CAD compared to individuals without CAD in this and other research (Xu et al., 2019), and though limited, some data suggest that this difference may be particularly evident among men (Xu et al., 2019). Though additional research examining sex differences in the association between CAD and LTL is needed, it is plausible that the shorter (and less variable) LTL in men with CAD in the current study compared to the other groups may have precluded additional effects of childhood maltreatment, in essence creating a floor effect on LTL in men with CAD. Given the paucity of data on the potential moderating role of health status and sex, further research is warranted to replicate our findings.

The relation between childhood maltreatment and LTL was not found to be moderated by the various markers of emotional dysregulation (anxiety, depression, stress), but perceived stress over the past two years was found to partially mediate this relation. This is consistent with various theories that have proposed that the experience of childhood maltreatment may lead to a selective search for threat, difficulties perceiving social and environmental cues of safety, as well as overactive fight-flight-or freeze physiological and behavioral responses to these threats (Porges, 2009; Thayer & Lane, 2000). In essence, a psychobiological context is created in which victims of childhood maltreatment are chronically perceiving, experiencing, and perhaps contributing to stress. According to the Hidden Talent Theory (Ellis et al., 2017), these alterations were likely adaptive for a child exposed to constant threat because they helped promote immediate survival (e.g. social withdrawal in the context of abuse may help prevent unwanted attention) and temporarily reduce acute distress, but may hold long-term health costs when maintained. In the current sample, significantly greater symptoms of anxiety, depression, and chronic stress were reported by participants who experienced maltreatment in childhood indicating that such emotional dysregulation may persist well into older age. The precise mechanisms by which chronic stress or other markers of emotional dysregulation mediates the impact of childhood maltreatment on LTL have not been delineated, though poor health behaviors (in attempts to self-regulate negative mood; Khantzian, 1997) and repeated solicitation of fight-flight-freeze responses and associated autonomic, metabolic, endocrine, and immune changes have been proposed to be involved (Porges, 2009; Thayer & Lane, 2000). More specifically, it has been suggested that these recurrent physiological changes in response to perceived threat contribute to greater allostatic load (McEwen & Stellar, 1993) and dysregulation of mitochondrial structure and function, and as a result altered TL regulation (Juster, Russell, Almeida, & Picard, 2016). Further research is planned to test whether telomere attrition in survivors of childhood maltreatment is explained by increased allostatic load.

Several limitations of this study bear mention. The measure of childhood maltreatment was retrospective and self-reported and could have been influenced by memory bias (Baldwin & Danese, 2019). The cross-sectional nature of the study does not allow for causal inferences to be made. While prospective research is obviously required for mediation analyses, the current results nonetheless contribute to our understanding of the factors that may contribute to shorter LTL following childhood maltreatment. Results may not be generalizable to other ethnicities considering the majority of participants were French-speaking Caucasians and race/ethnicity differences in TL have previously been reported in the literature (Brown, Needham, & Ailshire, 2017; Ly et al., 2019). Although not examined within the current study, it is possible that differences in the chronicity and age at onset of maltreatment, or genetic vulnerability may have confounded the results (White & Kaffman, 2019).

The strengths include the use of validated questionnaires, a large sample size with a representative sample of men and women by which sex differences could be examined and validated qPCR LTL measurement methods that have been validated by our group in several studies (e.g. Buscarlet et al., 2017; Mollica et al., 2009; Starnino, Busque, Tardif, & D'Antono, 2016; Starnino et al., 2021). To our knowledge, this is the first study to examine the potential moderating and mediating role of psychological distress in the relation between childhood maltreatment and LTL relation among older individuals with chronic illnesses. The examination of three indices of emotional dysregulation is clinically useful, as each index has been shown to reflect different types of maladaptive emotional regulation strategies. For example, suppression may be more frequently used in the case of anxiety (Amstadter, 2008) as compared to rumination in the case of depression (Joormann & Gotlib, 2010).

In conclusion, childhood maltreatment predicted accelerated biological aging, as measured by LTL independently of patient characteristics among older individuals, though this effect was less obvious in men with CAD. Chronic stress resulting from childhood maltreatment may contribute to this process. Whether stress management interventions in survivors of childhood maltreatment could slow or lower their risk for premature ageing requires investigation. Such treatments have been shown to alter telomere regulation processes in a variety of samples (Schutte & Malouff, 2014), and to reduce risk for clinical events in individuals with established cardiovascular disease (Blumenthal et al., 2016).

## Supplementary material

For supplementary material accompanying this paper visit https://doi.org/10.1017/S0033291722003543. click here to view supplementary material
