# Supplementary material for: Childhood maltreatment and leukocyte telomere length in men and women with chronic illness: an evaluation of moderating and mediating influences
Source: Psychol Med. 2022 Nov 24;53(13):6242–52. doi: 10.1017/S0033291722003543 (PMC10522448; doi:10.1017/S0033291722003543)
Supplement: Supplementary file 1 [file S0033291722003543sup001.docx]

**Supplementary Table 1**

*Number of Biological Years Difference Between Those Exposed to Childhood Maltreatment and Those Not Exposed*

|  | Relative telomere length (T/S ratio) | Number of base pairs (bp) | Years difference in biological age (exposed vs not exposed) |
| --- | --- | --- | --- |
| **Men with CAD** |  |  |  |
| Exposed to childhood maltreatment | 0.844 | 5310.572 | Less than 1-month difference |
| Not exposed to childhood maltreatment | 0.845 | 5312.985 |  |
| **Women with CAD** |  |  |  |
| Exposed to childhood maltreatment | 0.866 | 5363.658 | 1.5 to 5-years |
| Not exposed to childhood maltreatment | 0.929 | 5515.677 |  |
| **Non-CVD men** |  |  |  |
| Exposed to childhood maltreatment | 0.832 | 5281.616 | 1.5 to 5-years |
| Not exposed to childhood maltreatment | 0.896 | 5436.048 |  |
| **Non-CVD women** |  |  |  |
| Exposed to childhood maltreatment | 0.890 | 5421.570 | 7-months to 2 years |
| Not exposed to childhood maltreatment | 0.915 | 5481.895 |  |
| **All participants** |  |  |  |
| Exposed to childhood maltreatment | 0.858 | 5344.354 | 11-months to 3-years |
| Not exposed to childhood maltreatment | 0.896 | 5436.048 |  |

*Note*. Years difference in biological age is an imprecise estimate based on the existing literature suggesting a 30-100 base pair reduction per year (Aviv, Valdes & Spector, 2006; Harris et al., 2016; Müezzinler, Zaineddin, & Brenner, 2013). The mean number of base pairs for each group was calculated using the formula (TL(kb) = 3.274 + 2.413 (T/S)) described by The Centers for Disease Control and Prevention, 2012.

**References**

Aviv, A., Valdes, A. M., & Spector, T. D. (2006). Human telomere biology: Pitfalls of moving from the laboratory to epidemiology. *International Journal of Epidemiology*, *35*(6), 1424–1429. https://doi.org/10.1093/ije/dyl169.

Centers for Disease Control and Prevention. (2012). National Health and Nutrition Examination Survey: 2001–2002 data documentation, codebook and frequencies. Retrieved from https://wwwn.cdc.gov/nchs/nhanes/2001-2002/L40_B.htm.

Harris, S. E., Marioni, R. E., Martin-Ruiz, C., Pattie, A., Gow, A. J., Cox, S. R., … Deary, I. J. (2016). Longitudinal telomere length shortening and cognitive and physical decline in later life: The Lothian birth cohorts 1936 and 1921. *Mechanisms of Ageing and Development*, *154*, 43–48. https://doi.org/10.1016/j.mad.2016.02.004.

Müezzinler, A., Zaineddin, A. K., & Brenner, H. (2013). A systematic review of leukocyte telomere length and age in adults. *Ageing Research Reviews*, *12*(2), 509–519. https://doi.org/10.1016/j.arr.2013.01.003.
